# Managed Metapopulations: Do Salmon Hatchery ‘Sources’ Lead to In-River ‘Sinks’ in Conservation?

**DOI:** 10.1371/journal.pone.0028880

**Published:** 2012-02-08

**Authors:** Rachel C. Johnson, Peter K. Weber, John D. Wikert, Michelle L. Workman, R. Bruce MacFarlane, Marty J. Grove, Axel K. Schmitt

**Affiliations:** 1 Institute of Marine Sciences, University of California Santa Cruz, Santa Cruz, California, United States of America; 2 Chemical Sciences Division, Lawrence Livermore National Laboratory, Livermore, California, United States of America; 3 Anadromous Fish Restoration Program, United States Fish and Wildlife Service, Stockton, California, United States of America; 4 East Bay Municipal Utility District, Lodi, California, United States of America; 5 Southwest Fisheries Science Center, National Marine Fisheries Service, Santa Cruz, California, United States of America; 6 Department of Earth and Space Sciences, University of California Los Angeles, Los Angeles, California, United States of America; 7 Department of Earth and Space Sciences, University of California Los Angeles, Los Angeles, California, United States of America; University of Otago, New Zealand

## Abstract

Maintaining viable populations of salmon in the wild is a primary goal for many conservation and recovery programs. The frequency and extent of connectivity among natal sources defines the demographic and genetic boundaries of a population. Yet, the role that immigration of hatchery-produced adults may play in altering population dynamics and fitness of natural populations remains largely unquantified. Quantifying, whether natural populations are self-sustaining, functions as sources (population growth rate in the absence of dispersal, λ>1), or as sinks (λ<1) can be obscured by an inability to identify immigrants. In this study we use a new isotopic approach to demonstrate that a natural spawning population of Chinook salmon, (*Oncorhynchus tshawytscha*) considered relatively healthy, represents a sink population when the contribution of hatchery immigrants is taken into consideration. We retrieved sulfur isotopes (^34^S/^32^S, referred to as δ^34^S) in adult Chinook salmon otoliths (ear bones) that were deposited during their early life history as juveniles to determine whether individuals were produced in hatcheries or naturally in rivers. Our results show that only 10.3% (CI = 5.5 to 18.1%) of adults spawning in the river had otolith δ^34^S values less than 8.5‰, which is characteristic of naturally produced salmon. When considering the total return to the watershed (total fish in river and hatchery), we estimate that 90.7 to 99.3% (CI) of returning adults were produced in a hatchery (best estimate = 95.9%). When population growth rate of the natural population was modeled to account for the contribution of previously unidentified hatchery immigrants, we found that hatchery-produced fish caused the false appearance of positive population growth. These findings highlight the potential dangers in ignoring source-sink dynamics in recovering natural populations, and question the extent to which declines in natural salmon populations are undetected by monitoring programs.

## Introduction

Many species across a diversity of taxa exhibit metapopulation structure where the persistence of species depends on their existence as sets of local populations, largely independent yet interconnected by dispersal (e.g., insects, birds, fish; reviewed in [1]). Thus, understanding the scale and extent of dispersal among populations has been central to conservation efforts aimed at minimizing species' risks of extinction on increasingly fragmented or isolated landscapes [2], [3]. Managing for optimal levels of dispersal adds an additional level of complexity since it is one of the most difficult demographic parameters to quantify, especially for migratory species.

Species-specific life history characteristics related to dispersal and gene flow are central to successful recovery and conserving efforts [1]. For example, the evolution of philopatry contributes to the highly variable life history patterns and genetic diversity characteristic of many salmonids by facilitating local adaptation. Many fitness-related traits are heritable in salmonids fishes [4], allowing spatial variation in selection to drive the adaptive divergence of reproductively isolated populations (reviewed in [5]). In contrast, straying, or the tendency of adults to return to breed at sites other than their birthplace, tends to reduce reproductive isolation. Such demographic subsidies can be critical for reducing population extinction risks in unproductive populations [1], [6]. Thus, the frequency and extent of straying (e.g., dispersal) from the natal spawning grounds defines the demographic and genetic boundaries of a population and is central to the persistence of salmon populations.

The metapopulation concept and theoretical source-sink dynamics driven largely by habitat quality has proven useful for understanding population structure of Pacific salmon *Oncorhynchus spp.* [7]–[9]. However, no empirical studies to date have demonstrated the occurrence of source-sink dynamics in salmon, let alone identified the consequences that occur when the source population is a managed subsidy. Identifying populations or habitats that function as sinks (e.g., λ<1) is difficult because of the challenges in measuring key demographic parameters [10]. Sinks are therefore doomed to extinction if not rescued by immigrants from a source population where reproduction is greater than mortality. Therefore, populations functioning as sources provide a critical link to the long-term persistence of a metapopulation (population of populations, sensu Levins [11]) through numerical contribution of individuals within their local population and as immigrants to sink populations [10]. Quantifying immigration (or straying) of adult salmon into non-natal locations to spawn has been impeded largely by the inability to identify their birthplace.

The use of adult census data, in conjunction with an ability to detect immigrants is an extremely valuable tool for the conservation and management of populations. For example, when source-sink dynamics exist, the abundance of a species in an area can be disconnected from the specific survivorship and fecundity rates of that area, owing to the presence of individuals produced from other areas. The disconnection between abundance and population productivity poses at least three potential problems in conservation: (1) sink populations may be perceived as self-sustaining, while actually relying on immigration from source populations without which they could become extinct [7], [9], [12], (2) if population abundance is no longer a good indicator of habitat quality or habitat productivity, one could conserve the wrong type of habitat [10], [13], and (3) attempts to relate abundance to habitat characteristics (e.g., habitat restoration actions) may mask the presence of a sink habitat or potentially overestimate restoration efficacy [8], [9], [14].

The role of artificial propagation in recovering threatened and endangered populations to sustainable levels is one of the most controversial issues in applied ecology [15]. Recently, conservation hatcheries that raise and release modest numbers of hatchery fish have been adopted as a tool for reintroductions, to reduce inbreeding depression, and to maintain a lineage for stocks that are near extinction in the wild. Yet, production hatcheries that release enormous numbers of hatchery-produced fish to enhance in-river produced salmon stocks remains the cornerstone of salmon conservation and harvest management worldwide [14]–[19]. Recent concern has been raised as to whether production hatcheries aimed at producing fish for harvest may compromise fitness and thus recovery of imperiled populations in the wild [16], [17]. Regardless of whether the presence of hatchery-produced fish has a negative impact on natural salmon populations, reproduction by hatchery fish presents an accounting problem that complicates the estimation of population growth rates. This occurs because the natural in-river population is being supplemented by an external population (the hatchery).

The concept that unmarked hatchery-produced fish spawning in natural rivers may mask declines in natural populations is not new [18], [19]. Hatchery production of seven species of salmonids throughout the north Pacific from Japan to the west coast of the United States has increased dramatically [20]. However, empirical data does not exist to quantify the extent to which hatchery-produced fish are spawning “naturally” in rivers because the majority of hatchery-produced fish are unmarked and thus cannot be identified on spawning grounds.

In this study, we use the natural variation in sulfur isotopes in fish otoliths (ear bones)- to reconstruct and quantify (1) the unknown proportion of hatchery-produced Chinook salmon spawning in-river, (2) the contribution of hatchery immigrants to a watershed's total return (river and hatchery), and (3) spawning habitat associations by hatchery and naturally-produced salmon. We use these data and additional demographic criteria to identify for the first time salmon populations functioning as sources and sinks. We also highlight the need to identify and quantify hatchery immigrants (via physical or natural markers) in adult censuses to understand natural reproduction and survival- data necessary and lacking for conservation and hatchery management.

## Materials and Methods

### Ethics statement

All work was conducted in accordance to animal welfare guidelines approved by the University of California, Santa Cruz Chancellor's Animal Research Committee (permit Barnr0811).

### 2.1 Sulfur isotopes in otoliths

Natural variation in elemental chemistry of otoliths is becoming a fundamental tool in fish ecology to reconstruct habitat origin and track movement of individual fish (reviewed in [21]). Elements are permanently imbedded within otoliths and can be measured from discrete daily growth increments deposited throughout the life of a fish. Many of these elements, such as strontium, have been shown to come primarily from the water [22], [23] and therefore their concentrations and/or their isotopic ratios, ^87^Sr/^86^Sr, can be effective watershed markers [24]–[26]. Here we use sulfur isotopes (^34^S/^32^S, δ^34^S), which enter the otolith's protein matrix from dietary sources [27] to differentiate between habitats with different foodwebs, such as marine versus fresh water [28], or in our case, residence in a river versus a hatchery. The aquatic food web is significantly different in hatchery facilities than in rivers [29]. Hatchery salmon feed has high δ^34^S values (+14.1‰ to +16‰) because the majority of the protein in the fish meal comes from the marine fish tissues (+17‰ to +18‰). Wild juveniles feed on freshwater aquatic insects which typically have lower δ^34^S values (∼Ο±10‰; [29]–[31]). The differences in food webs are detected in the otoliths of hatchery and naturally-produced fish and can be measured in adults to reconstruct the differences in rearing sources experienced as juveniles, with little to no ambiguity [29]. If δ^34^S were combined with a watershed marker, such as strontium isotopes [24]–[26], both watershed and rearing habitat could be determined; but a watershed marker was not used in this study.

### 2.2 Salmon study system

Like many salmonids globally, several of California's native salmonids are in an unprecedented decline and are at risk of extinction [32]. Until recently, Chinook salmon that spawned in the fall in California's Central Valley rivers (USA) were considered healthy populations. Several factors have been implicated in their recent population decline with the role of freshwater insult, ocean conditions, and hatcheries chief among them. Today, hatchery-produced fall-run salmon dominate the system nine-to-one [33] and the low numbers of adults returning to spawn has resulted in the consecutive closure of salmon fishing off the California and Oregon coasts for the first time in its 100 year history.

The Mokelumne River is one of the largest salmon producing rivers for fall-run Chinook salmon in California. While not all of California's salmon producing streams have hatcheries associated with them, rivers with hatcheries are the largest producers of salmon in the state. The Lower Mokelumne River supports populations of Chinook salmon and steelhead (*O. mykiss*), both of which are the subject of long-term and on-going monitoring and restoration efforts [34]. Natural in-river production is supplemented by artificial production by the Mokelumne River Fish Hatchery (hereafter ‘hatchery’ or MRFH), which has been in operation since 1964 and releases 4–10 million juvenile Chinook salmon annually [35].

**Figure 1:**
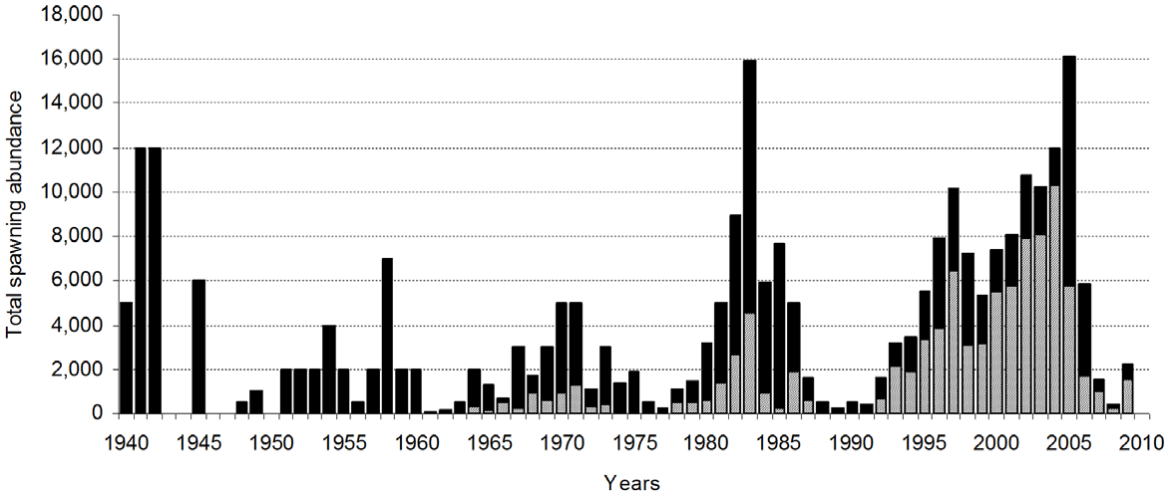
Adult Chinook salmon population trend. Stacked bar graph of the total number of adult fall-run Chinook salmon (*Oncorhynchus tshawytscha*) spawning on the Mokelumne River (black bars), and in the Mokelumne River Fish Hatchery (grey bars) from 1940–2009 (East Bay Municipal Utility District, unpublished data). Graph shows adult spawning location and not rearing origin. Note: Mokelumne River Fish Hatchery was built in 1964.

Natural juvenile fall-run Chinook salmon rear in freshwater typically for 3–6 months and migrate out through the Sacramento-San Joaquin Delta and into the ocean where they typically spend 1–3 years before they return as adults to spawn from September through December and complete their lifecycle [36]. Hatchery fish are released either in-river or into the Sacramento-San Joaquin Delta during the same period as natural emigration. The average number of total spawning adults returning to the Mokelumne River watershed (returns to the river plus the hatchery) has ranged from 410 fish in 1991 to over 16,000 fish in 2005 (Fig. 1).

### 2.3 Otolith collection and preparation

**Figure 2:**
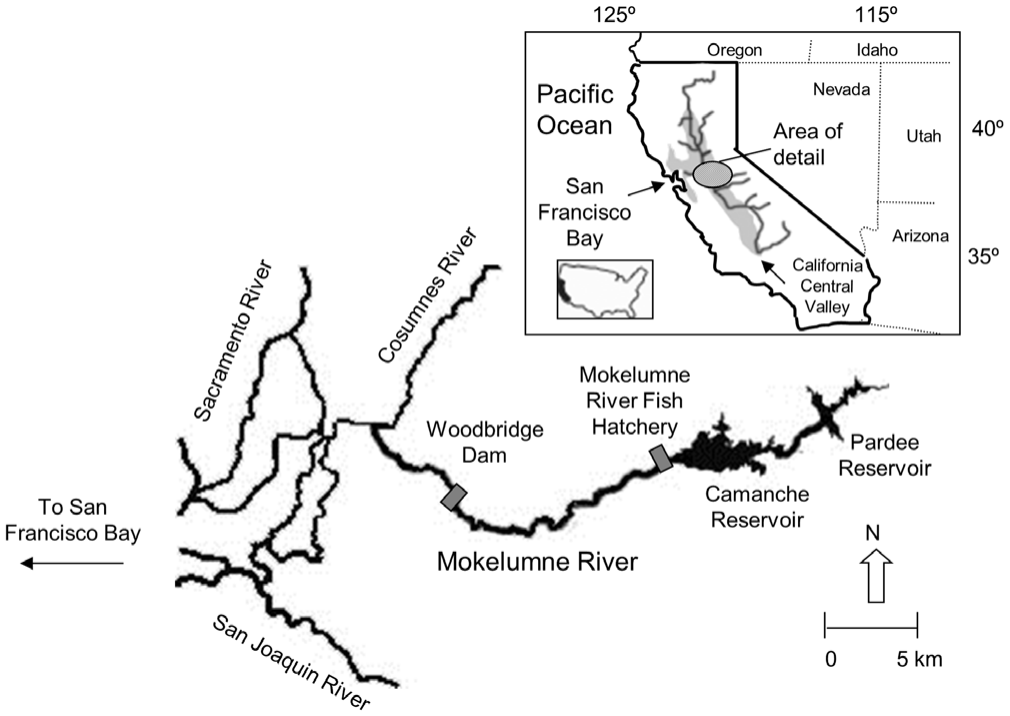
Map of study region. The Mokelumne River and Mokelumne River Fish Hatchery in relation to the western United States, and the Sacramento-San Joaquin river system (shaded region) in California's Central Valley (insert). The in-river spawning habitat on the Mokelumne River consists of the area between its confluence with the Cosumnes River and upstream to the Camanche Dam (∼16 km).

Otoliths were collected from adult Chinook salmon returning to spawn at the hatchery (N = 947) and in-river (N = 363) during carcass surveys by biologists for the East Bay Municipal Utility District between October 2004 and January 2005 (Fig. 2). Otoliths were also collected from known-origin hatchery adults (N = 13) determined by recovery of coded wire tags (CWTs) implanted into fish as juveniles at the hatchery. Otoliths were extracted in the field, rinsed, and stored dry until mounting. A subset of these samples were randomly selected and provided without collection information for analysis to address each of our primary objectives.

Otoliths were first aged whole, embedded in epoxy and polished on both sides to reveal internal structure necessary for isotopic analysis of the juvenile growth portion of the otolith [29], [37]. Otoliths were polished so that the daily growth bands after the exogenous feeding check were clearly visible, transferred individually on 1-inch diameter glass rounds, and polished flush with the surface of the surrounding epoxy exposing the otolith core.

### 2.4 Sulfur isotope analyses

Sulfur isotopic analysis was performed in otoliths using the method of Weber et al. [29]. The otoliths were analyzed in two sessions by secondary ion mass spectrometry (SIMS) at the University of California, Los Angeles, W.M. Keck Foundation Center for Isotope Geochemistry using an ims1270 (CAMECA). For both sessions, the primary ion current was ∼1.5 nA Cs^+^ with Köhler illumination producing a ∼30 µm analysis spot on the sample representing an average of 6–10 days of otolith growth. The secondary ions ^32^S^−^ and ^34^S^−^ were collected primarily in peak hopping mode using a single axial electron multiplier (EM) for the first session and static collection mode (using two multi-collector EMs) for the second session.

To determine the origin (hatchery or river) of each individual fish, δ^34^S was measured (∼3 analysis spots) in the otolith corresponding to deposition just after juveniles completed feeding from maternal yolk and began feeding exogenously (post exogenous feeding or PEF), but before out-migration [33]. When the exogenous feeding check was indistinct on the otolith, analyses were conducted between 250 and 400 µm from the center of the otolith. These PEF analyses were compared to δ^34^S analyses (∼3 spots) of the otolith region deposited prior to exogenous feeding (core) or after marine entry (margin), which should represent marine influenced δ^34^S values. This internal standardization method increases measurement precision because the absolute value of δ^34^S in the PEF can vary between otolith mounts due to shifts in Instrument Mass Fractionation (IMF) rather than differences exclusively due to feeding ecology. This can occur because of differences in beam alignment after exchanging samples, and differential deterioration of electron multiplier gains in multi-collection analysis. For one session, the variation in the δ^34^S analyses of the marine portion of all analyzed otoliths exceeded the precision of the individual measurements by ∼50% (2SD = 5.7‰ vs. 2SE∼4‰, respectively; SE = standard error). In some cases, internal referencing was not used because the core value was later determined to be obscured by non-core otolith material. In these cases, the PEF data were referenced to the average marine δ^34^S value found in the marine growth region of other otoliths. Uncertainty in the average marine δ^34^S value is based on the overall variability in these measurements.

### 2.4.1 Data reduction

Because the absolute sulfur isotopic composition of otoliths has not been established, the PEF data are standardized based on estimates of the isotopic composition for the core and marine regions of growth. The marine growth region of the otolith is assumed to have δ^34^S_CDT_ = 18‰, the isotopic composition of adult salmon tissue [38]. In this study, we found that the marine region differs in isotopic composition from the core by 4.6±1.1‰ (mean ±2 SE) based on replicate paired core and margin analyses. These data are used to correct the PEF data relative to CDT. For the purposes of assigning individuals to rearing origin, this approach does not reduce accuracy because the relative difference between hatchery and river δ^34^S does not change. First, uncorrected ratios, (^34^S/^32^S)_raw_, from the ion probe measurement were converted to δ^34^S values in parts permil (‰) relative to CDT (^34^S/^32^S)_CDT_ = 0.044163 [39] using:
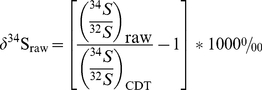
(1)Note that δ^34^S_raw_ is different from the true value because of instrumental mass fractionation (approximately −20‰), and therefore should not be regarded as an accurate value relative to CDT. In cases where an internal reference was available, PEF δ^34^S data were corrected for IMF using the relation:
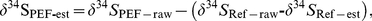
(2)where δ^34^S_PEF-est_ is the estimated PEF δ^34^S value relative to CDT, δ^34^S_Ref-est_ is the estimated δ^34^S value for the reference region relative to CDT, and δ^34^S_Ref-raw_ and δ^34^S_PEF-raw_ are the mean uncorrected δ^34^S values for the reference and the PEF regions, respectively. Standard error propagation is performed by summing the standard errors for the mean δ^34^S values for PEF, internal reference, and mean of the reference region in quadrature. This error estimate is for total internal error across samples and does not take into account the uncertainty in the absolute CDT δ^34^S value of the reference regions, which is discussed below. For PEF δ^34^S measurements without internal reference analyses, the data are corrected for the mean IMF estimated from the marine measurements:
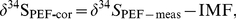
(3)where IMF = δ^34^S_Ref-meas_−δ^34^S_Ref-est_. For these PEF δ^34^S estimates, internal precision is estimated by summing in quadrature the standard error for the mean PEF δ^34^S value and the estimate of sample-to-sample measurement precision (SD of all marine measurements for the session).

### 2.4.2 Method validation

Otoliths from 13 CWT fish of known MRFH origin were analyzed along with the other otoliths in this study without knowledge of their identities to determine the accuracy of the sulfur isotope assignments. Further validation was conducted using otolith samples from juvenile Chinook salmon collected from the Mokelumne River (known natural-origin) and hatchery (known hatchery-origin). Otoliths from naturally-produced adult Chinook salmon from the spring-run on Butte Creek and winter-run on the Sacramento River, California and a juvenile from the Salmon River in Idaho of known-origins were also used to determine accuracy in assignments.

### 2.5 In-river estimate

**Figure 3:**
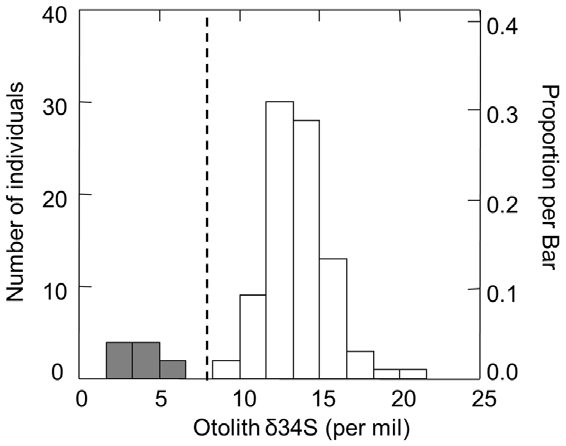
Frequency distribution of δ^34^S values in salmon otoliths. Histogram of otolith δ^34^S for the juvenile rearing portion of otoliths from unknown origin adult Chinook salmon (*Oncorhynchus tshawytscha*) spawning in-river on the Mokelumne River (USA). Fish assigned to natural origin (grey bars; N = 87) had δ^34^S values less than 6‰ (dashed line) and did not overlap with δ^34^S values from those identified as originating from a hatchery (open bars; N = 10). These results indicate that 90% of in-river spawners were produced in a hatchery.

To determine the rearing origins of Chinook salmon spawning in-river on the Mokelumne River, 97 otoliths from the carcass survey were selected, analyzed, and assigned to rearing origins using sulfur isotopes. Assignments were based on empirically determined cut off of δ^34^S = 8.5‰ for PEF in analysis of known origin Chinook salmon and observed break in the bimodal distribution of δ^34^S values between 5.5 and 8.9‰ (Fig. 3). The assignment cut-off of δ^34^S = 8.5‰ was chosen to allow for higher natural spawning values from other rivers in the California Central Valley. Individuals with PEF values above 8.5‰ were assigned hatchery-origin.

To achieve the most accurate point estimate of the proportion of fish originating from hatcheries, we used Laplace's procedure [40]. This approach has been determined to be more robust than dividing the number assigned to hatchery-origin by the total number of fish in the sample [41]. The 95% confidence interval (CI) for the population is governed by binomial statistics and was calculated using the Adjusted Wald estimate modified for small sample sizes [42].

### 2.6 Watershed level estimate

For the overall watershed level estimate (in-river plus hatchery), samples were selected in-proportion to the numbers of fish returning to the hatchery (N = 10 356) and spawning in-river (N = 1 588). We analyzed otoliths from 83 adult salmon from the hatchery and 12 collected from carcasses in-river to achieve this goal (N = 95). We used the assignment criteria described above to identify individuals to rearing origins and estimated their watershed level proportions to the spawning population using the Laplace point estimate and Adjusted Wald for 95% CI.

### 2.7 Habitat association

To determine whether there was an association between return location (e.g., in-river and hatchery) and production origin (naturally or hatchery-produced), we analyzed an additional 12 otoliths from adults that returned to the hatchery and 85 from in-river spawners. Using samples from the other project objectives achieved a balanced sampling design of 95 samples from adults returning to the hatchery and 97 spawning in-river. To assess habitat associations between rearing types, a two factor Chi-square test was conducted.

### 2.8 Population growth rate

Two estimates were made for population growth rates for Chinook salmon spawning in the Mokelumne River between 1992–2004. These years were chosen because of reliable in-river and hatchery spawning abundance data as well as juvenile production estimates from in-river spawners and hatchery releases. The first estimate, ‘apparent’ or ‘total’ population growth rate, λ_T_, refers to the growth rate of the Mokelumne River watershed salmon population irrespective of the proportion of hatchery or natural-origin spawners that returned to the hatchery or spawned in the river, and thus includes hatchery immigrants. The second estimate, ‘actual’ or ‘natural’ population growth rate, λ_N_, is an estimate of the population growth rate of natural spawners, which accounts for whether adults who survived to spawn in the watershed spent their early life in a hatchery or a river. Therefore, λ_N_ is an estimate of natural reproduction and survival without the influence of hatchery immigrants. Note that λ_T_ includes both hatchery and natural-origin fish, whereas λ_N_ is the growth rate for the natural population only.

Apparent population growth rate was calculated using an age-structured cohort replacement model. We estimated the number of individuals from each cohort that survived to spawn (apparent cohort survival for year t, S_T,t_) using the following relationship:
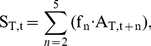
(4)where A_T,t+n_ is the total number of adult salmon returning to spawn in the river and the hatchery (naturally and hatchery-produced) in year t+n (n = age class 2, 3, 4, and 5 year olds). f_n_ is the fraction of the year class represented in returning adults in year t+n. Given the dearth of age data for this system, the age distribution was estimated from coded wire tag recoveries of adult salmon from Mokelumne River Fish Hatchery (MRFH) caught in the freshwater sport fishery, carcass survey, or at MRFH from 1990–1995 (Regional Mark Information System Database). Based on those data, f_2_ = 0.25, f_3_ = 0.62, f_4_ = 0.13, f_5_ = 0.004 Eq. 4 assumes that hatchery and in-river produced fish have the same age distribution, which has not been tested. The apparent growth rate is:
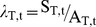
(5)For a cohort to replace itself, the value of λ_T_ must be 1. Populations with values of λ_T_<1 are in decline and values >1 indicate population growth.

Actual natural population growth rate, λ_N_ is the growth rate resulting from natural reproduction in the absence of hatchery individuals. To estimate the survival of the progeny of in-river spawners only, we included information on the proportion of natural-origin fish in the annual adult return data. This study provides an empirical estimate of this proportion for 2004. For earlier years where no empirical measures exist, we estimated the proportion of natural-origin fish (natural production to total juvenile production) for a given cohort based on the relative survival of natural and hatchery juvenile production found in this study. Natural production was estimated from rotary screw trap data on the Mokelumne River, and hatchery production came from an estimate of the number of fish released from the hatchery. We used the same age-structured cohort replacement model as outlined in our apparent growth rate analysis. However, the actual in-river cohort survival was estimated as:
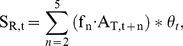
(6)where θ_t_ is an estimate of the proportion of natural-origin fish for year t.

As an estimate of θ_t_, we use the ratio of the naturally produced juvenile salmon emigrating from the river to the total number of juvenile salmon produced in the river and hatchery in year t. This approximation is intended as a proxy to capture the magnitude difference between the hatchery releases to in-river production as a null expectation if survivorship was equal. This approach is generally supported by our empirical findings in 2004. Our empirical point estimate of 4.1% (CI = 0.7 to 9.3%) natural-origin adults returning to the watershed is in close agreement (within error) to the weighted reconstruction of the natural production to total production for the four contributing year classes (1.3%).

The number of adults that return to the river to spawn, A_R,t_, is determined by river carcass surveys, and unlike A_T,t_, it does not include adults that return to the hatchery to spawn. A_R,t_ is used to estimate the growth rate of the actual natural population for year t, λ_N,t_, along with the natural cohort survival, S_N,t_:
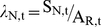
(7)λ_N,t_ is an estimate of natural reproduction and survival without the influence of hatchery immigrants.

We demonstrate the model's response to variation in θ_t_ by accounting for a factor of 3 increases in survival of natural-origin salmon. The difference we tested is based on the observed difference between our empirical estimates in 2004 when compared to our proxy. We achieved this by substituting 3θ_t_ for θ_t_ in eq. 6 and used that value for S_N,t_ in eq. 7 to calculate the population growth rate under this condition. Further, we calculate the difference in survivorship between naturally and hatchery-produced salmon required to result in λ_N_≥1. We solved for values of θ_t_ in eq. 6 that produced λ_N_≥1 using eq. 7 and then calculated the factor difference between our estimated proxy of θ_t_ using the juvenile production and hatchery releases and dividing it by the value of θ_t_ required for positive population growth.

## Results

### 3.1 Method validation

Our validation test using known-origin Chinook salmon (based on CWT) resulted in correct identification of all fish from the hatchery (13 of 13; 100% correct) without our prior knowledge of their identities. All δ^34^S values for the CWT fish were significantly greater than 8.5‰, with an average of 13.7‰ (SD = 1.9‰). In addition, 25 independent analyses were made for 21 Chinook salmon of known origin, all of which were correctly identified by otolith sulfur.

The accuracy for the sulfur isotope method was consistent with expectation. This method is based on the known difference between the sulfur isotopic composition of the hatchery diet, which is based on marine fish meal, and the freshwater prey items [29]. Therefore, the accuracy of identification is based on the accuracy of the sulfur analysis. Accuracy was maintained by analysis of known samples, internal standardization, and rerunning samples for which the initial analysis had low classification confidence, as well as random reruns. For the unknowns, a total of 29 out of 205 samples were rerun once, and two samples were rerun twice. Based on the high degree of separation in δ^34^S data (non-overlapping distributions), and accuracy in classifying known-origin samples, we conclude that population discrimination based on sulfur isotopes is robust.

### 3.2 Proportion of hatchery fish

Hatchery-produced fish comprised the vast majority of adult Chinook salmon spawning in the river and in the watershed while naturally-produced adults were largely absent (Table 1). We found that 89.7% of adults spawning in the river (CI = 81.9 to 94.5%) and 95.9% of adults spawning in the watershed (CI = 90.7 to 99.3%) in 2004 were produced in the hatchery based on the value of δ^34^S being greater than 8.5‰ in the PEF area of the otolith. Values of δ^34^S greater than 8.5‰ reflect a marine source of protein during freshwater rearing in the hatchery (Fig. 3). Our results indicate that only 10.3% (CI = 5.5 to 18.2) of fish spawning in the river and 4.1% (CI = .7 to 9.3%) of the fish spawning in the watershed were the progeny of in-river parents. The bimodal distribution in δ^34^S values with non-overlapping observations between 5.5‰ and 8.9‰ demonstrates the binary assignment of individuals to rearing origin using δ^34^S values.

**Table 1:** Estimate of hatchery and natural-origin salmon.

Spawning location	Hatchery			Natural		
	Laplace	Adjusted Wald 95% CI		Laplace	Adjusted Wald 95% CI	
		low	high		low	high
Watershed	95.9%	90.7%	99.3%	4.1%	0.7%	9.3%
In-river	89.7%	81.9%	94.5%	10.3%	5.5%	18.1%

Proportion of adult Chinook salmon (*Oncorhynchus tshawytscha*) spawning in-river on the Mokelumne River (USA) or within the entire Mokelumne River watershed (river+hatchery) assigned to hatchery or natural origins based on δ^34^S values in otoliths. Laplace point estimates and 95% Confidence Intervals (CI) were calculated using the Adjusted Wald estimate modified for small sample sizes.

A disproportionate number of fish returned to the location where they were produced. While the majority of fish spawning in the hatchery and in-river were of hatchery-origin, we found a positive association between production origin (hatchery vs. natural) and spawning location- MRFH vs. in-river. The sulfur isotope method detected a greater number of naturally produced fish spawning in-river (10 of 97) than returning to spawn at the hatchery (3 of 95; Chi-square, χ^2^ = 3.8, df = 1, p<0.05).

### 3.3 Population growth rate

**Figure 4:**
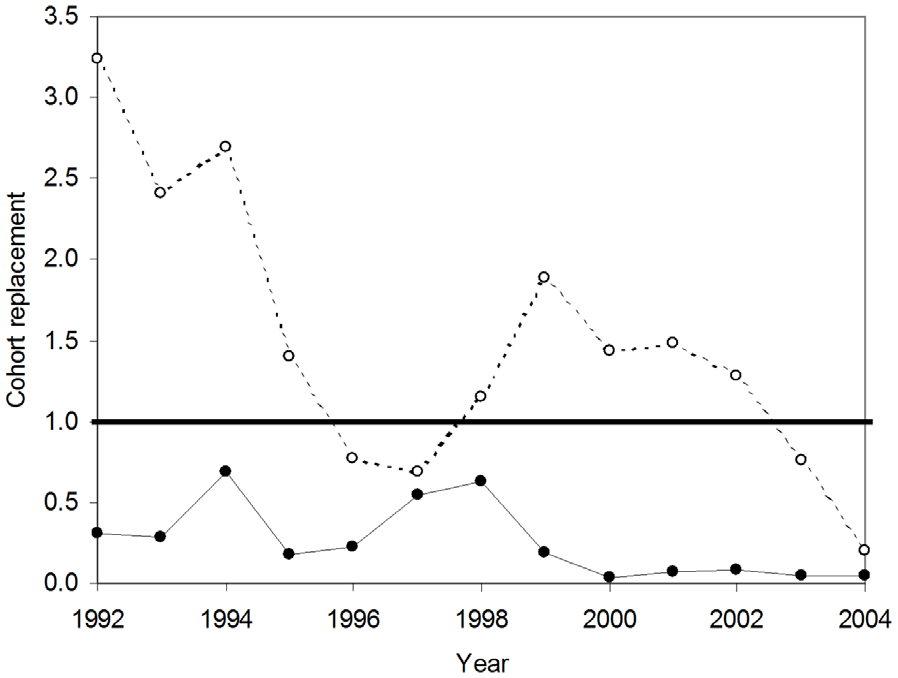
Population growth rates. Population growth estimates of Chinook salmon (*Oncorhynchus tshawytscha*) on the Mokelumne River watershed from cohort reconstruction. Apparent growth rate estimates (open circles) show several years where cohort replacement values exceed 1 (solid line). Natural population growth rates (filled circles) remove the influence of immigration from hatchery fish. These results suggest that in-river populations are being supported by hatchery sources.

Hatchery immigrants masked the lack of a viable natural population (Fig. 4). The apparent population growth rate for salmon was greater than 1 (e.g., positive population growth) during the majority of years (9 of the 12; average λ = 1.49; Fig 4). In the absence of hatchery immigrants, we estimate that the natural population would have exhibited population growth rates that are not sustainable; λ<1 in all 12 years (Fig. 4; Table 2).

**Table 2:** Cohort reconstruction and population growth rates.

Adult abundance			Adult Age Structure^1^				Apparent		Actual	
Year	Total (A_T_)	River (A_R_)	Age 2 (.25)	Age 3 (.62)	Age 4 (.13)	Age 5 (.004)	cohort survival (S_T_)	growth rate (λ_T_)	cohort survival (S_N_)	growth rate (λ_N_)
1992	1,645	935	405	1,022	212	7	5,330	3.24	288	0.31
1993	3,157	993	777	1,960	407	13	7,617	2.41	281	0.29
1994	3,421	1,503	842	2,124	441	14	9,219	2.69	1,029	0.68
1995	5,517	2,194	1,357	3,426	712	22	7,700	1.4	397	0.18
1996	7,920	4,037	1,948	4,918	1,022	32	6,077	0.77	928	0.23
1997	10,175	3,690	2,503	6,319	1,313	41	7,009	0.69	2,000	0.54
1998	7,213	4,123	1,774	4,479	930	29	8,292	1.15	2,573	0.62
1999	5,335	2,182	1,312	3,313	688	21	10,045	1.88	402	0.18
2000	7,418	1,894	1,825	4,607	957	30	10,611	1.43	74	0.04
2001	8,114	2,305	1,996	5,039	1,047	32	12,042	1.48	157	0.07
2002	10,757	2,844	2,646	6,680	1,388	43	13,725	1.28	222	0.08
2003	10,240	2,123	2,519	6,359	1,321	41	7,814	0.76	104	0.05
2004	11,944	1,588	2,938	7,417	1,541	48	2,450	0.21	100	0.06
2005	16,140	10,406	3,970	10,023	2,082	65	.	.	.	.
2006	5,871	4,139	1,444	3,646	757	23	.	.	.	.
2007	1,519	470	374	943	196	6	.	.	.	.
2008	412	173	101	256	53	2	.	.	.	.
2009	2,233	680	549	1,387	288	9	.	.	.	.

Cohort reconstruction and population growth estimates for Chinook salmon (*Oncorhynchus tshawytscha*) on the Mokelumne River (USA) for all returning adults (Apparent population growth rate) and natural origin spawners (Actual natural population growth rate).

1 Age structure determined by coded wire tag recoveries of adult salmon from Mokelumne River Fish Hatchery (MRFH) caught in the freshwater sport fishery, carcass survey, or at MRFH (RMIS database). The number of survivors from each cohort is the sum of age 2, 3, 4, and 5 year olds produced in a given spawning year (cohort survival). For example, the return data in the ‘Adult Age Structure’ columns that are in the bold cells sum to the apparent cohort survival value for 1992.

Results of our analysis on the response of actual population growth rates of the natural population to varying values of θ_t_ indicate that a broad range in values for the proportion of natural-origin spawners resulted in the same general finding. A factor of 3 differences in survivorship (3θ_t_), as observed between our empirical estimate in 2004 and this proxy, produces the same interpretation. In fact, our estimate of the proportion of natural-origin fish could vary on average by a factor of 10, with a minimum of 1.5, and still show negative population growth rates (Table 2). Our proxy assumption would have to differ by the numerical factors (factor diff θ_t_) to produce a stable population (λ_N_ = 1; Table 3).

**Table 3:** Percent natural-origin salmon and influence on population growth rate estimates.

Juvenile production				Response to variations in θ_t_		
					Parameters λ_N_ = 1	
Year	% natural (θ_t_)^2^	Natural	Total	growth rate^3^ (3θ_t_)	% natural^4^ θ_t_	Factor diff^4^ θ_t_
1992	5.4	183448	3,398,082	0.92	17.5	3.2
1993	3.7	143,224	3,887,370	0.85	13	3.5
1994	11.2	434,000	3,889,747	2.05	16.3	1.5
1995	5.2	184,014	3,566,462	0.54	28.5	5.5
1996	15.3	540,466	3,538,740	0.69	66.4	4.3
1997	28.5	1,848,539	6,478,611	1.63	52.7	1.8
1998	31	1,535,439	4,947,646	1.87	49.7	1.6
1999	4	168,525	4,213,525	0.55	21.7	5.4
2000	0.7	68,294	9,816,692	0.12	17.9	25.7
2001	1.3	77,346	5,924,666	0.2	19.1	14.7
2002	1.6	140,471	8,693,125	0.23	20.7	12.8
2003	1.3	87,654	6,573,568	0.15	27.2	20.4
2004	4.1	432,874	6,514,300	0.19	64.8	16.2
2005	.	1,197,778	6,539,113		.	.
2006	.	19,582	6,188,945		.	.
2007	.	18,347	4,660,647		.	.
2008	.	30,614	260,460		.	.
2009	.	.	.		.	.

2 Percent natural is the number of juvenile outmigrants estimated from rotary screw trap sampling relative to both natural and hatchery production in a given spawning year, except in 2004 where our empirical value from this study was used.

3 Population growth rate estimated using a factor of 3 increases in survival of natural-origin fish.

4 Calcualtion of the value of θ_t_ required to produce a stable population (λ_N_ = 1) and the factor difference between this value and our proxy. Note: Growth rate analysis could only be conducted through year 2004, as it requires 5 year classes to return and spawn.

## Discussion

The strong tendency of salmon to return to their natal rivers may be responsible for a vision of salmon spatial structure as a collection of nearly isolated populations [43]. Thus, a census of in-river spawners may be thought of as adequate in determining whether natural populations are self-sustaining. This indirect measure and perception can lead to erroneous conservation actions when hatchery production supplements the total return. For example, we found that the abundance of Chinook salmon spawning in the river was substantially disconnected from the specific survivorship and fecundity rates of naturally produced fish owing to immigrants from a hatchery source. Adult spawning abundance in the absence of knowledge of rearing-origin, produced a false sense of river productivity and survivorship, a central tenant of many restoration objectives. The potential discrepancy between in-river spawning abundance and natural production may be particularly important in years when natural population abundances are critically low.

Without an ability to identify hatchery from natural-origin fish, declines in natural populations could be more widespread than is currently recognized. For example, the Columbia River Basin, USA has a significant number of production hatcheries and is one of the most data-rich salmon watersheds, yet a census of spawning adults in-river (a composite of both natural and hatchery-reared spawners) is used to assess population status and viability in over half of their stocks [18], [44]. This underscores the paucity and importance of empirical data to inform conservation and recovery actions for threatened and endangered stocks and those important to fisheries.

The natural population of Chinook salmon spawning in the river is a demographic sink. Mortality of juveniles produced by adults spawning in-river is higher than their parental generation in all 12 years from 1992–2004. However, immigration from the hatchery ‘source’ masks this lack of sustainability in 9 of 12 years. Our empirical findings using δ^34^S suggest that while an estimated 11 944 fish returned and spawned in the watershed in 2004, only 10.3% (CI = 5.5 to 18%) of 1 588 in-river spawners were produced there (N = 87 to 286) and only 4.1% (CI = 0.7 to 9.3%) of the total spawning population were of natural-origin (N = 84 to 1 111). Based on the cohort reconstructions, the mortality of the juveniles produced from the 1,588 in-river spawners in 2004 was severe, showing low cohort survival (N = 73; Table 2).

Several factors likely contribute to the low survivorship of the natural population resulting in its reliance on external subsidy for persistence. Low survival could be due watershed degradation, water diversions, pollution, overfishing, oceanic conditions, precipitation, predation, food availability, and hatcheries [45]. Hatchery fish immigrating and spawning in-river can function to increase overall population size and may be necessary for preventing the extinction of some in-river populations. Yet, this dynamic is particularly complex because hatchery immigrants may also be a factor contributing to low survivorship of the natural population. There is mounting evidence that hatchery fish differ from their wild counterparts on a variety of life history, genetic, behavioral and demographic characteristics (reviewed in [46], [47]) and that introgression of hatchery-selected genes may diminish fitness in the wild [48]–[50]. Hatchery fish may indirectly exacerbate the negative population growth rate for the next generation of juveniles produced in the wild [51]. This creates a challenge in managing the resource- the hatchery may be functioning as a critical conservation tool that itself may erode the natural population. Work by Araki and colleagues [52] show experimental evidence that significant population declines in steelhead trout can be caused by reduced fitness (40% per captive-reared generation) when hatchery fish spawn in-river. Recently, reproductive performance in Chinook salmon was also found to be negatively associated with the proportion of hatchery spawners [53]. We found that juveniles that are produced and rear in-river are likely 1–2 generations removed from hatchery parentage based on our results that the vast majority of in-river spawning adults were hatchery-produced fish. If the same fitness effects are applicable to Chinook salmon in California's rivers, then these hatchery impacts could be a significant component to the observed larger-scale population decline.

While we fully acknowledge the limits of our cohort reconstruction, these limits do not invalidate the fundamental conclusion that based on our stable isotope data, naturally-produced fish account for only a fraction of in-river production. There is equivocal empirical support for survivorship differences between hatchery and natural-origin fish. Survival of hatchery fish has been reported to be lower [54], [55], higher [21], [51], [52] or similar [56] to that of wild fish. Regardless of these uncertainties in the estimated proportion of natural salmon for prior years, we are confident that because the natural production is so out of balance with the population that we are able to reach these conclusions with only limited data. If we would have assumed hatchery fish had greater survival, which has been shown in other systems, the outlook for the natural population is worse. Further, our method produces a conservative estimate of the hatchery influence as it only considers the current generation. For example, if two hatchery fish spawn in the river, they will produce an offspring with a “natural” signature despite the fact that their parents were produced in the hatchery. This limitation of our method points to an even greater impact of hatchery fish than our results suggest. Overall, many more fall-run Chinook salmon spawn in the rivers than in the hatcheries in the California Central Valley [57]. However, the number of successfully outmigrating juveniles per spawning adult in the rivers is not well documented, but is clearly large [57]. This fundamental observation has resulted in a census of in-river spawners referred to as “natural” being the metric for in-river viability used to manage ocean harvest. Without knowledge about the production origin of individuals, no assessment can be made on the sustainability of natural populations. In systems like the Mokelumne River where source-sink dynamics exist between hatcheries and in-river populations, in-river census can lead to the erroneous perception of a sustainable natural population.

Hatchery releases have increased over time in California [57]. A comparison of estimates of the contributions of hatchery fish to fall-run populations suggests that the proportion of hatchery fish in the system has similarly increased and, in fact, that hatchery fish may be replacing natural fish. Barnett-Johnson et al. [33] showed by otolith analyses that 90±6% (1 SD) of 158 Chinook taken in the ocean fishery off Central California in 2002 originated from hatcheries. We found in this study that in 2004, 97% (88–98% CI) of spawning adults on the Mokelumne River were of hatchery origin. These data together suggest that hatchery production is playing a significant role in California's salmon population dynamics.

A deeper understanding is needed regarding the extent to which the numeric supplementation from the hatchery is currently a pre-requisite for population persistence for the next generation of returning adults to in-river habitat. We found that natural-origin fish, although numerically small, spawned preferentially in the in-river habitat by a factor of 3. This preference has been found on small-scales in other salmon systems [58]. This suggests that if there is an accompanying preference of wild-origin fish to mate with other wild-origin individuals, then co-evolved gene complexes may still remain within the natural population and could become reestablished with reduced immigration from hatchery sources and decreased mortality.

In this system, the hatchery is functioning as a numeric ‘source’ providing a large number of immigrants to the natural spawning population on the Mokelumne River. Indirectly, they could be contributing to demographic sinks if they reduce population fitness in-river. Several key aspects of immigration and emigration are still poorly understood and need to be examined in detail on relevant scales to salmon conservation. These include the spatial and temporal scales of movement, the origin and destination populations instead of simple straying rates, the relative reproductive success of immigrants and residents, and how these factors are influenced by whether an immigrant is of hatchery or natural origin.

Understanding the associations between trends in abundance and demographic processes such as survivorship and immigration is fundamental for conservation and management. Identifying the extent to which abundance is decoupled from the viability of local populations is particularly important. Production of hatchery salmon baring no identifying marker to their origin has reached global scales [20], and thus source-sink dynamics between natural populations and hatchery-produced fish has not been adequately monitored. As the societal, ecological, and political debates surrounding salmon conservation continue, our study provides a cautionary tale of the dangers of ignoring source-sink dynamics in salmon conservation.
